# Real-Time Non-Invasive and Direct Determination of Lactate Dehydrogenase Activity in Cerebral Organoids—A New Method to Characterize the Metabolism of Brain Organoids?

**DOI:** 10.3390/ph14090878

**Published:** 2021-08-30

**Authors:** Gal Sapir, Daniel J. Steinberg, Rami I. Aqeilan, Rachel Katz-Brull

**Affiliations:** 1 Department of Radiology, Hadassah Medical Organization and Faculty of Medicine, Hebrew University of Jerusalem, Jerusalem 9112001, Israel; gal.sapir1@mail.huji.ac.il; 2 The Concern Foundation Laboratories, The Lautenberg Center for Immunology and Cancer Research, Department of Immunology and Cancer Research-IMRIC, Hebrew University-Hadassah Medical School, Jerusalem 9112001, Israel; daniel.steinberg@mail.huji.ac.il (D.J.S.); ramiaq@mail.huji.ac.il (R.I.A.); 3 The Wohl Institute for Translational Medicine, Jerusalem 9112001, Israel

**Keywords:** organoids, dissolution dynamic nuclear polarization, lactate dehydrogenase, [1-^13^C]pyruvate

## Abstract

Organoids are a powerful tool in the quest to understand human diseases. As the developing brain is extremely inaccessible in mammals, cerebral organoids (COs) provide a unique way to investigate neural development and related disorders. The aim of this study was to utilize hyperpolarized ^13^C NMR to investigate the metabolism of COs in real-time, in a non-destructive manner. The enzymatic activity of lactate dehydrogenase (LDH) was determined by quantifying the rate of [1-^13^C]lactate production from hyperpolarized [1-^13^C]pyruvate. Organoid development was assessed by immunofluorescence imaging. Organoid viability was confirmed using ^31^P NMR spectroscopy. A total of 15 organoids collated into 3 groups with a group total weight of 20–77 mg were used in this study. Two groups were at the age of 10 weeks and one was at the age of 33 weeks. The feasibility of this approach was demonstrated in both age groups, and the LDH activity rate was found to be 1.32 ± 0.75 nmol/s (n = 3 organoid batches). These results suggest that hyperpolarized NMR can be used to characterize the metabolism of brain organoids with a total tissue wet weight of as low as 20 mg (<3 mm^3^) and a diameter ranging from 3 to 6 mm.

## 1. Introduction

In the past decade, the fields of developmental biology and disease modelling were revolutionized by the emergence of organoid cultures [1,2,3]. Organoids are now used for studying cancer [4] and various organ development and disease (e.g., kidney [5], liver [6], and pancreas [7]), as well as neural development and diseases [8]. Human brain organoids are derived from human pluripotent stem cells (hPSCs), which can be induced to form a wide variety of brain regions, such as the forebrain, midbrain, and hindbrain [9,10]. Metabolism is a key feature of embryogenesis and cellular disease. Due to the dynamic nature of organoid development and the fast and regulated changes that occur in them, real-time monitoring of enzyme activities in these research tissues could be beneficial. Considering the high level of resources needed for organoid preparation, it appears that a metabolic investigation that is non-invasive and non-destructive is especially needed. However, to the best of our knowledge, such enzymatic activities have not been explored in any tissue organoid preparation so far.

^13^C-NMR of hyperpolarized substrate metabolism [11] using the product-selective saturating excitation approach [12] allows real-time detection of instantaneous changes of in-cell metabolic enzyme activities. The most widely used hyperpolarized agent so far is [1-^13^C]pyruvate, due to its favorable chemical, physical, and biochemical/biological properties [13]. It is also important to note that studies with hyperpolarized [1-^13^C]pyruvate are translational as it is the lead agent for clinical use [13]. One of the most observed metabolic conversions when administering hyperpolarized [1-^13^C]pyruvate to mammalian tissues in vivo or ex vivo is the production of hyperpolarized [1-^13^C]lactate, which is due to the in-cell activity of lactate dehydrogenase (LDH) [14,15,16,17,18,19,20,21,22]. Previously, the metabolic properties of organoids were studied mostly by mass spectroscopy-based methods [23,24,25]. NMR was also utilized to examine metabolic properties in organoid lysates [26]. To the best of our knowledge, no studies were performed on viable intact organoids using NMR spectroscopy.

One of the main limitations in studying metabolism in cerebral organoids (COs) and organoids in general is their small size, as growth in these tissues is limited by diffusion. Small tissue size limits the hyperpolarized metabolites signal that could be detected, and the larger the organoid, the more the arrival of the hyperpolarized substrate to the entire body of viable cells in the organoid, to enable real-time metabolic monitoring at a temporal resolution of seconds, may be limited by diffusion. Nevertheless, as the organoids grow and the core becomes necrotic, most of the proliferation of cells takes place in the outer rim, up to approximately 500 µm from the surface [27]. This observation makes the outer rim the most relevant area in which to study metabolism, while this area is also the most likely to quickly encounter hyperpolarized substrates in the perfusate. As per these inevitable limitations and potential promise, we hypothesized that real-time metabolism of [1-^13^C]pyruvate could be determined in COs aged 10 to 33 weeks, which consisted of a sub-population of COs that was available for this study (grown in-house for up to 8 months). Here, we show that tissue metabolism in real-time can be investigated in organoids with a total wet weight as small as 20 mg and as large as 77 mg, and a diameter that ranged from 3 to 6 mm. To the best of our knowledge, this is the smallest intact tissue size from which metabolic NMR in real-time has been sampled, as regards tissues with multiple cell types and 3D structure, whether excised from mammals or produced as a mimic of a mammalian tissue. Furthermore, our approach maintained tissue viability, allowing for immunofluorescent studies to take place after hours of recordings in the NMR spectrometer.

## 2. Results

### 2.1. Validation of Cerebral Organoids Cellular Composition

**Figure 1:**
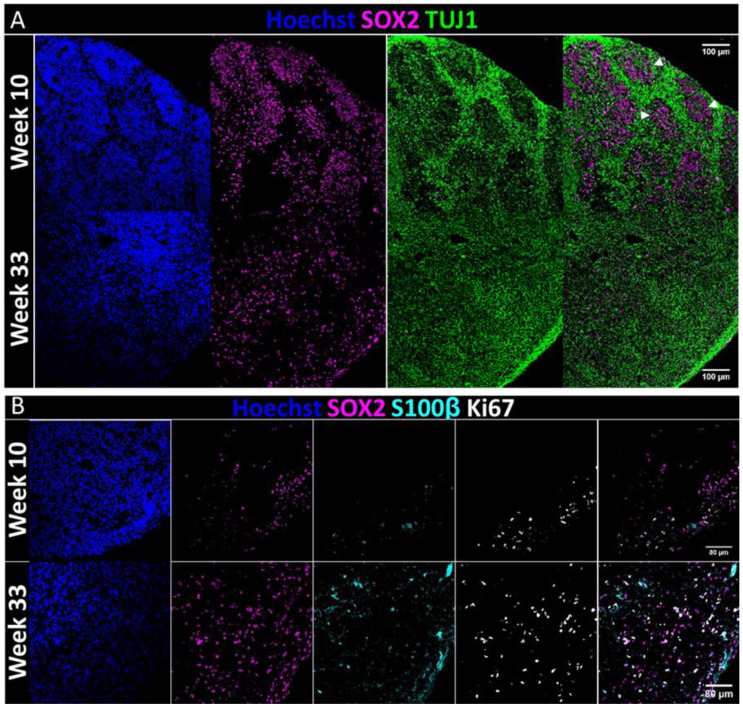
COs’ validation. (**A**) Validation of neural cellular development in the organoids: Immunofluorescent staining of weeks 10 and 33 COs, demonstrating the expression of Neuron-specific class III β-tubulin (TUJ1) and the neuronal progenitors’ marker SOX2, and the formation of a ventricular-like zone (VZ, white arrowheads), which is lost at week 33 (Week 10: n = 4, Week 33: n = 4). White bar is 100 μm. (**B**) Validation of the presence of glial cells in the organoids: Immunofluorescent staining of Weeks 10 and 33 COs, validating the presence of glial cells (marked by S100β) and their ongoing proliferation in the COs throughout the culture period (marked by Ki67), arising mainly from SOX2+ cells (Week 10: n = 4, Week 33: n = 4). White bar is 80 μm. Hoechst—staining for cellular nuclei.

In order to validate the successful generation of COs, the organoids were fixated and cryosectioned at weeks 10 and 33 and stained for general neuronal markers and SOX2, which is a nuclear marker for pan-radial glia (the progenitor cells of the central nervous system). The cells formed a rosette-like structure, resembling the developing ventricle, which was previously termed a ventricular-like zone [9] (VZ, Figure 1A, white arrowheads). This structure was surrounded by cells positive for class III β-tubulin (TUJ1), a marker of early-born neurons, occupying an area resembling the developing cortical plate (Figure 1A). Through the culture period, as seen at week 33, the VZ structure was lost, in agreement with previous descriptions [28] (Figure 1A). To examine the presence of non-neuronal population, we stained for S100 calcium-binding protein β (S100β), an astrocytic marker, demonstrating a gradual increase in the prevalence of astrocytes through maturation. To validate the proliferation capabilities of the cells we stained for Ki67, which demonstrated active proliferation at both time points. We note that almost all Ki67-positive cells are SOX2-positive cells. The SOX2-positive cells outside the VZ are called outer radial glia (oRGs) and are particularly interesting, as they are abundant in the human brain and are considered a major source of human neurogenesis. Further characterization can be found in the Supplementary Materials S4. It is important to note that week 33 immunostaining was performed on COs after performing the NMR studies, suggesting that this technique holds long-term viability. Overall, the organoids demonstrated the presence of major neural populations and hallmarks of brain development.

### 2.2. Organoids Remain Viable in the NMR Spectrometer

**Figure 2:**
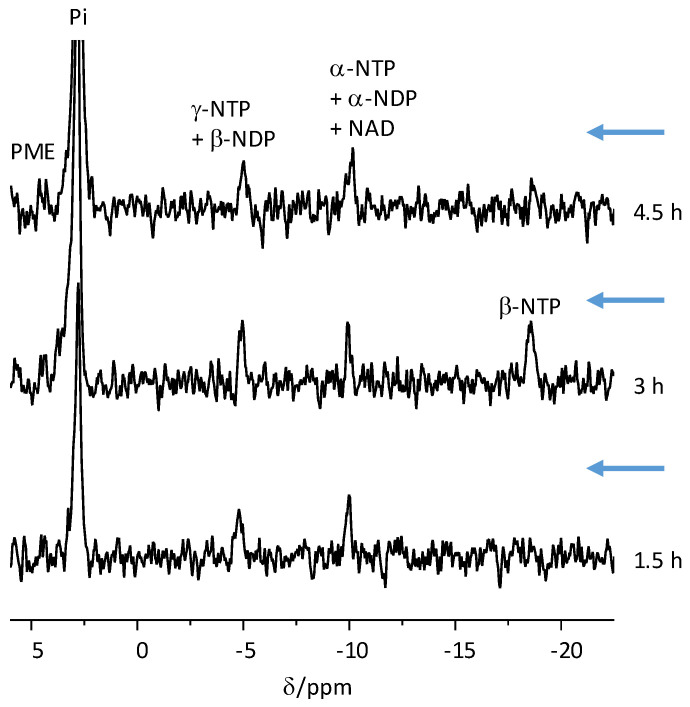
^31^P spectra of COs. ^31^P NMR spectra acquired from COs at 10 weeks. Acquisition of the first spectrum shown here began 0.5 h after the organoids were placed in the NMR tube. The time scale marks the end of the acquisition of each spectrum (t = 0 is the time when the organoids have finished recovery in the NMR spectrometer). The chemical shift was referenced to the α-NTP signal at −10.03 ppm. Each spectrum shown here was acquired with 4920 excitations (approximately 1.5 h). Pi, inorganic phosphate; NTP, nucleotide triphosphate; NDP, nucleotide diphosphate; NAD, nicotinamide adenine dinucleotide. The arrows indicate the times at which hyperpolarized [1-^13^C]pyruvate was injected, (immediately at the end of the acquisition of the ^31^P spectrum shown), and metabolism was recorded. The signal of inorganic phosphate (Pi) is truncated to better demonstrate the high energy phosphate signals. The signal of Pi increases after the injections due to incomplete washing of the hyperpolarized medium (which contains high Pi concentration).

The ^31^P spectra of COs showed the characteristic signals of nucleotide triphosphate (NTP), inorganic phosphate (Pi), and phosphomonoesters (PME), confirming their viability in the NMR spectrometer for more than 4.5 h (Figure 2). To the best of our knowledge, this is the first ^31^P NMR spectrum recorded from COs and organoids in general. Although brain spectra usually also show a phosphocreatine signal, this signal was not observed here. Most likely, this observation is related to the lack of creatine supplementation in the growth media. In the live mammal, PCr is generated from creatine, which is either supplied by the diet or synthesized de novo by enzymes in the kidney and liver.

### 2.3. Determination of LDH Metabolic Rate in Cerebral Organoids

**Figure 3:**
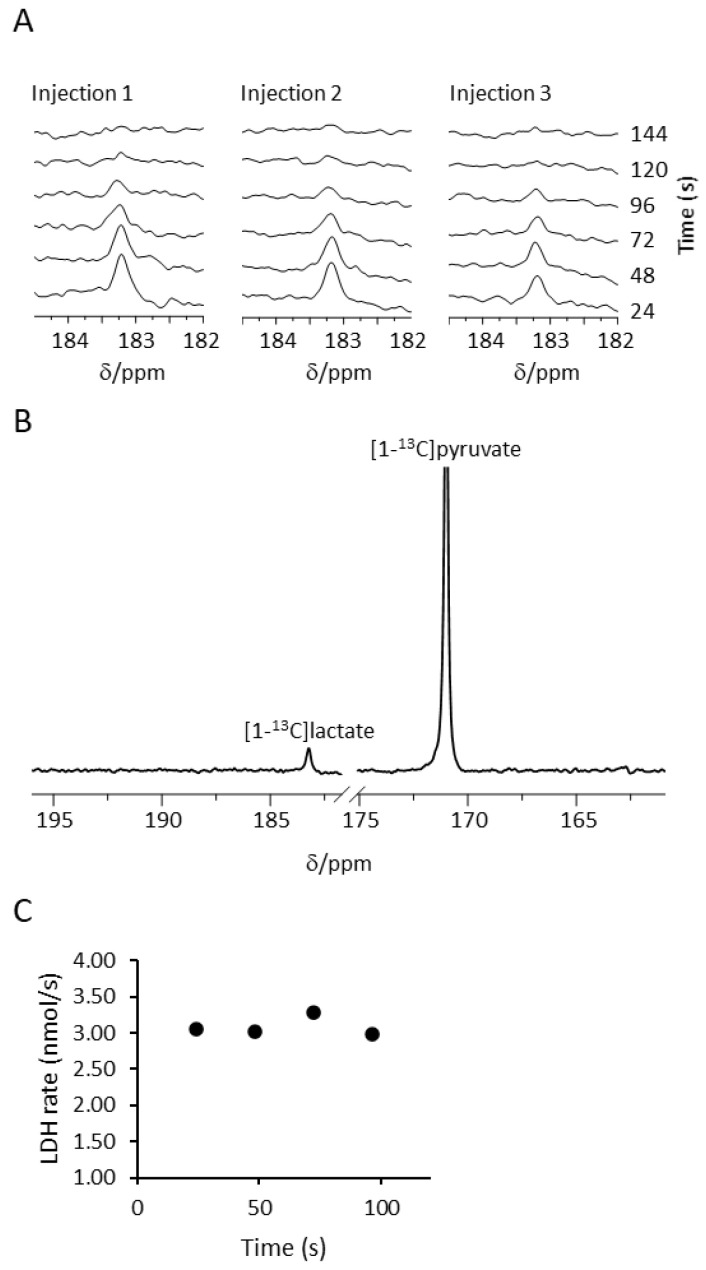
^13^C spectra of hyperpolarized [1-^13^C]pyruvate and LDH rate determination. (**A**) Consecutive ^13^C spectra following an injection of hyperpolarized [1-^13^C]pyruvate to COs. The timescale represents time after the first hyperpolarized signal of [1-^13^C]pyruvate appeared. Each spectrum represents the sum of three acquisitions. Spectral processing consisted of 7 Hz exponential line-broadening, 5% drift correction, and manual baseline and phase correction. The chemical shift was referenced to the [1-^13^C]pyruvate signal at 171 ppm. (**B**) A ^13^C spectrum, acquired following injection of hyperpolarized [1-^13^C]pyruvate to COs. The sum of 10 acquisitions from injection 1 is shown for a clear presentation of the [1-^13^C]lactate signal with respect to the [1-^13^C]pyruvate signal. Spectral processing was the same as in A. The signal of [1-^13^C]pyruvate is truncated to better visualize the [1-^13^C]lactate signal. (**C**) LDH rate over time following the same injection of hyperpolarized [1-^13^C]pyruvate shown in A (injection 1). Only points for which the signal-to-noise ratio for lactate was sufficient and for which the concentration of [1-^13^C]pyruvate was determined to be constant (Supplementary Materials S2 and S3) were included in the calculation.

After validating their viability in the NMR spectrometer, we proceeded to investigate real-time metabolism in the COs. Hyperpolarized [1-^13^C]pyruvate was injected directly above the organoids residing in the NMR tube and filled the medium that engulfed the organoids (see Supplementary Materials S1 for the location of lines in the NMR tube and S2 and S3 for flow characteristics) and ^13^C spectra were recorded. Figure 3A shows consecutive ^13^C spectra in a typical experiment with the signal of [1-^13^C]lactate observable at similar intensities on all three injections. In Figure 3B, the [1-^13^C]lactate signal is shown relative to the [1-^13^C]pyruvate signal. Figure 3C shows quantitative analysis of LDH activity during one of the injections (Injection 1, Figure 3A). To quantify the enzymatic rate, the data were acquired with the product selective saturating-excitation approach [12]. Only time points for which the concentration of [1-^13^C]pyruvate was constant (and therefore known) were used for rate determination (Supplementary Materials S2 and S3). The LDH activity rate was found to be 1.32 ± 0.75 nmol/s (average ± standard deviation, n = 3 experimental days). The entire dataset with COs’ information and enzymatic rates per injection is provided in the Supplementary Materials (Table S1).

## 3. Discussion

COs are a powerful tool in the study of neurological diseases, as they recapitulate key milestones of brain development, allowing easier genetic manipulation than in vivo models, and maintain the developmental timeline of the tissue that characterizes whole animal models. Brain organoids are increasingly studied, but, to the best of our knowledge, real-time metabolism in brain organoids has not been studied before.

Here, we used a combined approach of ^31^P and hyperpolarized ^13^C NMR spectroscopy to monitor the metabolism of viable intact COs in real-time. The organoids remained viable in the NMR spectrometer for more than 5 h, as indicated by the presence of NTP in their ^31^P NMR spectra. Additionally, the conversion of hyperpolarized [1-^13^C]pyruvate to [1-^13^C]lactate by LDH was demonstrated in COs in two timepoints in their development, including a sample containing as little as three organoids with a total wet weight of as low as 20 mg. Three injections were performed in each experiment. This may be beneficial in future studies, as pharmacologic or other interventions can be applied in the early stages of the experiment (e.g., following the first injection) and their effects can be studied over the course of hours. Further studies in the future are needed to validate the enzyme activity quantification and evaluate its reproducibility and accuracy.

As we were able to perform additional immunofluorescent studies on the organoids that underwent metabolic analysis in the NMR, it is possible that this system could allow for long-term tracing of metabolic development in the same organoids. COs were studied in two chosen timepoints. In week 10, the VZs are still defined but the COs already contain some mature neurons and astrocytes. By week 33 the VZ is lost, and the progenitor pool is diminished considerably. This late timepoint was added to investigate the potential for long-term follow-up of COs’ metabolism. In both timepoints, real-time metabolism was demonstrated. In future studies we aim to include 2–4-week-old COs.

LDH is often used as a marker for malignancy [29]. In glioblastoma multiforme (grade IV glioblastoma), one of the most aggressive brain tumors, LDH expression is considered important for disease progression [30]. It has been demonstrated that hyperpolarized ^13^C imaging shows the conversion of [1-^13^C]pyruvate to [1-^13^C]lactate in patients with brain tumors [31]. The current results suggest that in the future, it may be possible to use COs for the development of diagnostic imaging markers or therapeutics. The current findings warrant further investigations, comparing abnormal organoids (malignant or otherwise) to normal ones. As the described experimental system is not limited to COs specifically, other previously described organoid disease models can be envisioned [9,32].

Other studies have investigated small tissue sizes and small cell culture samples before. Of note, Patra et al. [33] have studied a single cancer spheroid sample at 13.9 T using thermal equilibrium ^1^H-NMR. Real-time metabolite content was observed but the temporal resolution for this experiment was tens of hours. In the current study, we have observed metabolic enzyme activities in real-time with a temporal resolution of a few seconds with more than twofold lower magnetic field strength (5.8T). Sriram et al. [34] have detected intra- and extracellular hyperpolarized [1,2-^13^C]lactate production in renal cell carcinoma cell lines of varied aggressiveness. In this study, the cells were packed into alginate microspheres and a homogenous mixture of 200–250 μL of these spheres was used for metabolic monitoring using hyperpolarized [1,2-^13^C]pyruvate at 11.6 T. In the current study, the volume of the COs was up to an order of magnitude smaller than the volume of these spheres, and the magnetic field strength was twice as low. Using a dedicated low-field NMR device, Jeong et al. [35] were able to record hyperpolarized metabolite signals from a cell suspension sample with as little as 10^5^ cells, in a net detection volume of 2 μL [35]. In these three studies (Patra et al. [33], Sriram et al. [34], and Jeong et al. [35]), the investigatory system consisted of a single cell line at a time, as opposed to an intact tissue which consists of multiple cell types, cellular interactions, and 3D structure as the COs that were used in the current study. In the current study, the NMR detection was not optimized for small volumes (detection within a 10 mm NMR tube in a low-field NMR spectrometer). It appears likely that the above advancements in small volume detection could potentially improve the detection of metabolism in organoids and allow even smaller sized single organoids to be investigated in the future.

## 4. Materials and Methods

### 4.1. Chemicals, Reagents, and Tissue Culture

The OX063 radical (GE Healthcare, Chalfont Saint Giles, UK) was obtained from Oxford Instruments Molecular Biotools (Oxford, UK). [1-^13^C]pyruvic acid was purchased from Cambridge Isotope Laboratories (Tewksbury, MA, USA).

DMEM-F12 (catalog number: 01-170-1A), MEM non-essential amino acids (NEAA, catalog number; 01-340-1B), sodium pyruvate (catalog number; 03-042-1B), penicillin-streptomycin (catalog number; 03-031-113), trypsin type C (catalog number: 03-053-1B), USDA certified hESCs-quality fetal bovine serum (FBS), neurobasal medium (catalog number: 06-1055-110-1A), human recombinant insulin (catalog number: 41-975-100), HEPES buffer (catalog number: 03-025-1B), and PBS (catalog number: 02-023-1A) were purchased from Biological Industries (Beit Ha’Emek, Israel). Knockout Serum Replacement (KOSR, catalog number: 10828-028), GlutaMax (catalog number; 35050-038), N-2 supplement (catalog number: 17502048), B27 supplement without vitamin A (catalog number: 12587010), and B27 supplement containing vitamin A (catalog number: 17504044) were purchased from Gibco (Waltham, MA, USA). bFGF (catalog number: 100-18B) was purchased from Peprotech (Rocky Hill, NJ, USA). Rho-associated kinase inhibitor (ROCKi, also known as Y27632, catalog number: 10005583) was purchased from Cayman Chemical Company (Ann Arbor, MA, USA).

Dispase II solution (catalog number: D4693), 2-mercaptoethanol (catalog number: M3148), heparin (catalog number: H3149), and vitamin C (catalog number: A4403), were purchased from Sigma-Aldrich (Rehovot, Israel). Ultra-low attachment 96-v-well plates (catalog number: MS-9096VZ) were purchased from S-Bio Prime (Hudson, NH, USA). Culture dishes of 90 mm (catalog number: 825-090-15-017) were purchased from Miniplast (Ein Shemer, Israel). CHIR-99021 (catalog number: 1386) was purchased from Axon Medchem (Reston, VA, USA). Matrigel (catalog number: FAL356231) was purchased from Corning (Tewksbury, MA, USA). Immunofluorescence Mounting Medium (catalog number: s3023) was purchased from Dako (Glostrup, Denmark). Optimal cutting temperature (OCT) compound (catalog number: BN62550) was purchased from Bar Naor LTD (Petah Tikva, Israel).

### 4.2. Cell Culture

The WiBR3 human embryonic stem cells (hESC) were provided as a gift by Dr. Jacob Hanna (Weizmann Institute of Science). hESC were maintained in a 5% CO_2_ atmosphere on irradiated DR4 mouse embryonic fibroblasts (MEF) feeder layers in FGF/KOSR conditions: DMEM-F12 supplemented with 15% KOSR, 1% GlutaMax, 1% NEAA, 1% Sodium-pyruvate, 1% penicillin-streptomycin, and 8 ng/mL bFGF. The medium was replaced daily, and cultures were passaged every 5–7 days by trypsinization with trypsin type C. After passaging, the medium was supplemented with 10 µM ROCKi for the first 24–48 h to improve stem cell survival following the procedure.

### 4.3. Cerebral Organoid Generation and Culture

**Figure 4:**
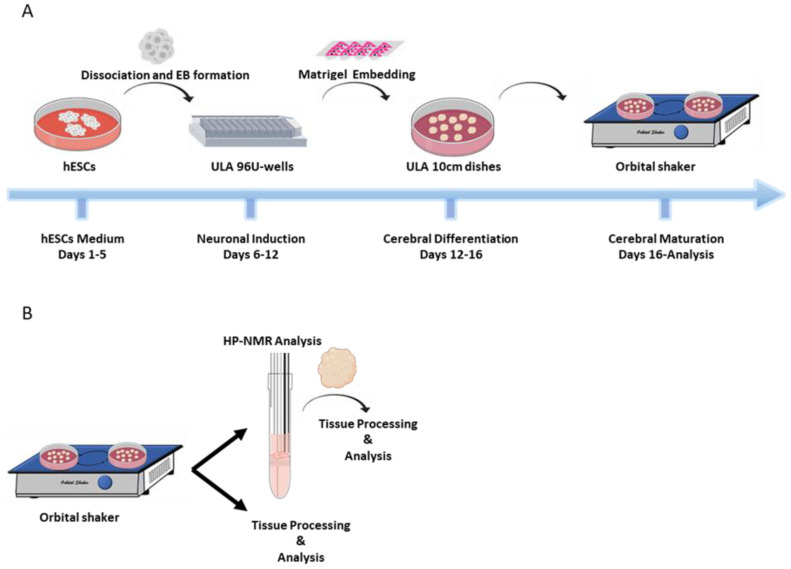
Generation of COs for analysis by hyperpolarized NMR and immunohistochemistry. (**A**) Illustration of the COs’ generation protocol. (**B**) Illustration of the COs’ analysis pipeline. ULA, ultralow attachment. EB, embryoid body. hESCs, human embryonic stem cells. HP, hyperpolarized.

COs were generated from the WiBR3 cells cultured as described above using a previously described protocol [32] (Figure 4A). Briefly, the WiBR3 cells were maintained on mitotically inactivated MEFs. Seven days before protocol initiation, the cells were trypsinized and counted. A total of 80,000 cells were seeded onto 60 mm plates coated with MEFs and grown until 70–80% confluence was reached. On day 0, intact colonies were detached from MEFs with 1 mg/mL Dispase II solution and dissociated to single cell suspension using a quick treatment with trypsin type C. Cells were then counted and suspended in hESCs medium, composed of DMEM/F12 supplemented by 20% KOSR, 3% FBS, 1% GlutaMax, 1% NEAA, 4 ng/mL bFGF, 10µM ROCKi and 100 µM 2-mercaptoethanol (the latter was added to prevent oxidation of essential components in the medium). For the generation of embryoid bodies (EBs), 10,000 cells were seeded in each well of an ultra-low attachment 96-v-well plates (Figure 4A). The medium of the EBs was replaced every other day for an additional 5 days, in which fresh bFGF and ROCKi were added only on the first medium change. On day 6, the EBs medium was changed to Neural Induction (NI) medium, composed of DMEM/F12, 1% N-2 supplement, 1% GlutaMax, 1% MEM-NEAA, and 1 µg/mL heparin solution. The NI medium was replaced every other day. On day 12, the EBs that successfully established neuroepithelium were embedded in Matrigel droplets [28]. Droplets were transferred to 90 mm sterile, nontreated, culture dishes with cerebral differentiation medium (CDM) composed of 1:1 mixture of DMEM/F12 and Neurobasal medium, 0.5% N-2 supplement, 1% B27 supplement without vitamin A, 1% GlutaMax, 1% penicillin/streptomycin, 0.5% NEAA, 50 µM 2-mercaptoethanol, 2.5 µg/mL human recombinant insulin and 3 µM CHIR-99021 (the latter is a WNT pathway activator, used here to support the acquisition of telencephalic identity in the COs). On day 16, organoids were cultured on an orbital shaker at 37 °C and 5% CO_2_ in cerebral maturation medium (CMM), which had a similar composition as CDM, with the following modifications: the B27 supplement was replaced by a B27 supplement containing vitamin A; addition of 400 µM vitamin C and 12.5 mM HEPES buffer; and without CHIR-99021. The medium was replaced every 2–4 days. From day 40 of the protocol, 1% Matrigel was added to the medium. To improve sterility, every 30 days the organoids were moved to fresh sterile plates. The media used for organoids culture were sterilized through a 0.22 μm filter before use. The COs (with a diameter of 3 to 6 mm) were collected at week 10 and 33 for further analysis via NMR and tissue processing for immunostaining (Figure 4B).

### 4.4. Solutions and Media for NMR Experiments

Solution 1: The medium used for continuous perfusion of the COs in the NMR spectrometer was the same as the CMM medium described above but without Matrigel.

Solution 2: A TRIS-phosphate buffer that was used for dissolution of the polarized sample contained 11.2 mM NaH_2_PO_4_, 38.8 mM Na_2_HPO_4_, and 50 mM TRIS. The dissolution buffer was titrated to reach a pH of 7.34–7.44 upon addition of 43 mM pyruvic acid.

Solution 3: 10 mL of Solution 1 together with 4 mL of Solution 2 containing the hyperpolarized [1-^13^C]pyruvate (formulation described below) made the hyperpolarized medium that was administered to the COs. The 10 mL of Solution 1 was bubbled with 95% O_2_/5% CO_2_ at a flow rate of 0.3 L/min (to prevent foam formation) for 5–9 min prior to the mix with Solution 2 and the administration to the NMR tube containing the COs.

### 4.5. Perfusion System and Administration of Hyperpolarized Medium to Cerebral Organoids

The perfusion of COs inside the NMR spectrometer and administration of the hyperpolarized medium to the COs was carried out as previously described for brain slices [15,21], with a few modifications required to allow continuous flow of perfusion media during hyperpolarized injections without any change to the position of the small COs inside the NMR tube. Upon transfer to the NMR tube, the COs were continuously perfused with medium at a flow rate of 4–4.2 mL/min. Between 80 and 100 mL of this medium was cycled between a reservoir bottle placed in a 40 °C water bath and the NMR tube. The reservoir was bubbled with humidified 95% O_2_/5% CO_2_ at a rate of 0.5 L/min to prevent foam formation for 1 h prior to COs’ perfusion and continuously bubbled with this gas mixture throughout the study. Inflow into and outflow from the NMR tube was delivered via medical grade extension tubes and pumped in a closed circle with a peristaltic pump (Masterflex L/S Analog Pump Systems, Cole-Parmer, IL, USA). The inflow and outflow lines were connected to thin polyether ether ketone (PEEK) lines. Inside the NMR tube, the organoids were placed on a custom-made filter (Porex, Interstate Specialty Products, MA, USA) which was held in the middle of the probe by a PEEK line (Supplementary Materials S1). This ensured that the COs were held in the sensitive zone of the NMR probe. An NMR-compatible temperature sensor was fixed inside the NMR tube, for monitoring the temperature throughout the experiment (Osensa, Burnaby, BC, Canada). The temperature in the NMR tube inside the spectrometer was kept at 33.6–36.9 °C, using heated air flow in the spectrometer and by keeping the inflow line in a heating blanket set to 40 °C (see also comment on temperature monitoring and variations in the Limitations section). An NMR compatible oxygen sensor (PreSens Precision Sensing GmbH, Regensburg, Germany) was also fixed inside the NMR tube and showed a level of 61.8 ± 3.4% (mean ± standard deviation, n = 3) oxygen saturation throughout the experiments. We performed the injections of hyperpolarized [1-^13^C]pyruvate using a bypass constructed of medical grade tubing and 3-way valves to obtain constant perfusion of the oxygenated hyperpolarized medium. For the injections, Solution 3 was pressure-injected into the bypass line which was submerged in a 40 °C water bath. The length of the bypass tube was adjusted to allow intake of 12 mL of Solution 3. That is, 2 mL of Solution 3 was ejected into a bin to ensure that the solution which would be pumped into the NMR tube was freshly oxygenated hyperpolarized medium, without any air bubbles which would interfere with the spectral acquisition. After pressure-filling the bypass line with hyperpolarized medium, the 3-way valves controlling inflow from the perfusion system to the bypass and outflow from the bypass into the NMR tube were opened. In this state, the peristaltic pump was able to push the hyperpolarized medium out of the bypass and into the NMR tube containing the organoids at a constant flow rate of 4 mL/min.

### 4.6. Experimental Workflow

A COs batch comprising 3–7 organoids (Supplementary Materials, Table S1) were transferred to an NMR tube with circulating perfusion medium, where they were maintained for the remainder of the experiment. Following 0.5 h of recovery in the NMR tube within the spectrometer, ^31^P spectra were acquired for at least 1.5 h for viability monitoring. The first injection of hyperpolarized [1-^13^C]pyruvate was then delivered and ^13^C spectra were acquired during the flow of the hyperpolarized medium through the NMR tube containing the organoids. Following the injection, ^31^P acquisition was resumed. This process was repeated for up to 3 times (up to 3 hyperpolarized injections per experimental day, Table S1).

### 4.7. DNP Spin Polarization and Dissolution

Spin polarization and fast dissolution were performed in a dissolution-DNP (dDNP) spin polarizer (HyperSense, Oxford Instruments Molecular Biotools, Oxford, UK) operating at 3.35 T. Microwave frequency of 94.132 GHz was applied for the polarization of a [1-^13^C]pyruvic acid formulation at 1.40–1.48 K. The formulation included 14 mM OX063 radical and 0.7 mM Gd^3+^ (as gadoterate meglumine, Dotarem, Guerbet, France). The amount of [1-^13^C]pyruvic acid formulation placed in the polarization cup was 14.48–15.35 mg. Following polarization, this formulation was dissolved in 4 mL of dissolution buffer (Solution 2). The dissolution was ejected into a conical tube which contained 10 mL of Solution 1, using a 6 s of He (g) chase to form Solution 3 (which was administered to the NMR tube containing the organoids).

### 4.8. Determination of Organoid Nucleotide Triphosphate Content

The nucleotide triphosphate content of the organoids was determined by integration of the γ-nucleotide triphosphate (NTP) signal, observed by thermal equilibrium ^31^P NMR spectroscopy (4920 averages) prior to each injection, and a comparison to an ATP standard of known concentration (0.1 M). This standard was made in-house and scanned separately on each experimental day. The γ-NTP signal of the COs was corrected for steady-state saturation using a T_1_ of 1.1 s, which was previously determined [36]. We note that the quantification of the NTP content in live tissues is based on the integration of the γ-phosphate of NTP because: (1) the α-NTP signal may contain NAD in addition to α-NTP, which would lead to overestimation of the NTP content; and (2) the β-NTP signal is usually wider due to interaction with Mg^+2^ ions and therefore its integration results in underestimation of the NTP content. In mammalian tissues, ATP is the predominant NTP species. However, in the spectroscopic conditions of perfused tissues it is impossible to resolve the particular NTP component. Indeed, it would also be hard to resolve the γ-NTP from β-NDP signal, and the α-NTP from the α-NDP signal. For this reason, these signals were assigned to both components (Figure 2). The NTP data are shown in Table S1.

### 4.9. NMR Spectroscopy

^31^P and ^13^C NMR spectroscopy were performed using a 5.8 T high resolution NMR spectrometer (RS2D, Mundolsheim, France), equipped with a 10 mm broad-band NMR probe.

### 4.10. ^31^P Spectroscopy

^31^P spectra were acquired with a repetition time of 1.1 s and a flip angle of 50°. Spectra were acquired in batches of 30 min (corresponding to 1640 excitations) and combined post-acquisition as needed.

### 4.11. Hyperpolarized ^13^C Spectroscopy

Hyperpolarized ^13^C data were acquired using the product-selective saturating excitations approach [12], applying 2.5 ms cardinal sine (sinc) pulses. Selective excitation for [1-^13^C]lactate and [^13^C]bicarbonate was applied consecutively and repeatedly with 4 s repetition time between pulses, yielding 8 s repetition time for each metabolite. For [1-^13^C]lactate acquisition the selective sinc pulse was centered 214 Hz upfield from the [1-^13^C]lactate resonance frequency, resulting in a [1-^13^C]pyruvate-to-[1-^13^C]lactate excitation ratio of 0.04. For [^13^C]bicarbonate acquisition the sinc pulse was centered 214 Hz down-field from the [^13^C]bicarbonate resonance frequency, yielding a [1-^13^C]pyruvate-to-[^13^C]bicarbonate excitation ratio of 0.12. A detailed description regarding the calibration of the RF pulses used in the current study has been previously published [17]. Using the selective product excitation, the hyperpolarized product signal was fully sampled upon each excitation and thus nulled prior to the next repetition time. This allowed for estimation of enzyme activity rate as described below.

### 4.12. Determination of LDH Activity

The LDH apparent enzymatic activity was calculated as follows. Due to the use of the product-selective saturating excitations approach [12], the signal of hyperpolarized [1-^13^C]lactate was fully sampled and depolarized by each selective pulse. This allowed for only newly synthesized [1-^13^C]lactate to be detected in the following excitation. The [1-^13^C]pyruvate signal is only minimally excited and is therefore almost not affected by the excitation pulses. To quantify the [1-^13^C]lactate production level, this minimally excited [1-^13^C]pyruvate signal was used as a reference. For quantification we selected a temporal window in which the [1-^13^C]pyruvate concentration was constant and therefore known (14 mM). This selection was based on the flow characteristics of the hyperpolarized medium through the NMR tube (Supplementary Materials S2 and S3). The signals of [1-^13^C]lactate that were obtained during this time window were used for the calculation of metabolite production rate using Equation (1):

(1) $$\upsilon_{lac}\left(t\right)=\left[pyr\right]*\frac{\rho \times {\mathrm{Vol}}_{pyr}}{\mathrm{TR}}*\frac{S_{lac}\left(t\right)}{S_{pyr}\left(t\right)}$$

where $\upsilon_{lac}\left(t\right)$ is the production rate of [1-^13^C]lactate at each time point, [pyr] is the maximal [1-^13^C]pyruvate concentration that could be delivered (14 mM), ρ is the selective pulse excitation ratio which was 0.04 for [1-^13^C]pyruvate to [1-^13^C]lactate, ${\mathrm{Vol}}_{pyr}$ is half of the volume occupied by the medium in the sensitive region of the NMR probe, as the Porex filter was placed in the middle of the probe (total volume estimated to be 1.375 mL), TR is the excitation interval for each metabolite which is 8 s, $S_{pyr}\left(t\right)$ is the signal of [1-^13^C]pyruvate at each time point, and $S_{lac}\left(t\right)$ is the signal of [1-^13^C]lactate at each time point. Data points with insufficient signal-to-noise ratio (<3) were excluded.

### 4.13. Spectral Analysis

Spectral processing and calculation of intensity integrals was performed using MNova (Mestrelab Research, Santiago de Compostela, Spain) and DMFit [37]. Statistical analysis was calculated with Excel (Microsoft, Ra’anana, Israel).

### 4.14. Immunofluorescence

COs’ fixation and immunostaining were performed as previously described [32]. The COs were washed in PBS and fixated in ice-cold 4% paraformaldehyde (PFA) for 45 min, which was followed by a wash in ice-cold PBS. For cryoprotection before embedding, the organoids were incubated overnight in 30% sucrose solution until equilibration was reached. The next day, the COs were embedded in OCT compound, frozen on dry ice and sectioned at 10 μm thickness using a Leica CM1950 cryostat. The sections were then stored at −80 °C until immunofluorescent staining. For immunofluorescent staining, the sections were warmed to room temperature for 15 min and washed in PBS for rehydration. Then, the sections were permeabilized in PBT (0.1% Triton X in PBS) for 10 min at room temperature. The permeabilized sections were submerged in blocking buffer containing 5% normal goat serum (NGS) and 0.5% BSA in PBT for 1 h to block nonspecific binding of antibodies. Following these steps, the sections were incubated overnight at 4 °C with primary antibodies diluted in blocking solution (Supplementary Materials, Table S2). The next day, the sections were washed 3 times with PBST (PBS containing 0.05% Tween-20) while shaking for 10 min. Then, the sections were incubated with secondary antibodies diluted in the blocking buffer for 1 h at room temperature (Supplementary Materials, Table S2). Nuclei were counter-stained with a 5 μg/mL Hoechst33258 solution. Slides were washed four times in PBST while shaking, and coverslips were mounted using an immunofluorescence mounting medium. Sections were imaged with an Olympus FLUOVIEW FV1000 confocal laser scanning microscope and processed using the device software.

## 5. Conclusions

The current results are a proof of concept that COs can be utilized for real-time metabolism observation using dDNP-NMR. This is critical, as the generation of organoids is a time- and resource-intensive process. In the future, we hope that the methods shown here for the metabolic investigation of COs can be used to study organoids of diverse tissue types and with various hyperpolarized tracers [38,39]. 

Limitations: In this study, the organoids were not further cultured after their measurements in the NMR spectrometer. However, by using a sterile perfusion apparatus, the same batch of organoids can be studied longitudinally, allowing better utilization of resources to obtain information regarding temporal changes in metabolism. As regards other metabolic pathways, in the current study we did not observe the conversion of [1-^13^C]pyruvate to [^13^C]bicarbonate, although it has previously been observed both in rat brain slices [15] and in the human brain [40]. This is possibly due to the miniscule amounts of tissue used here, leading to an insufficient signal-to-noise ratio of the [^13^C]bicarbonate signal, which is typically at least one order of magnitude lower than that of [1-^13^C]lactate. As regards the LDH activity that was monitored here, we note that this is a real-time in-cell activity, in the cells of the COs. Here, this activity had not been validated biochemically, as biochemical validation can only be conducted on homogenized tissues or cell lysates. Nevertheless, with this constraint in mind, this activity can be validated in the future and compared across developmental stages and pathological conditions. Additionally, we note that the concentration of glucose in the perfusion medium was relatively high (17.5 mM), as was the concentration of hyperpolarized [1-^13^C]pyruvate when administered to the COs (14 mM). Therefore, the observed metabolism may not recapitulate metabolism under different conditions. In the future, it may be warranted to include immunofluorescent staining of MCT and GLUT transporters.

Other technical limitations include the temperature range (33.6–36.9 °C), which may have an impact on metabolic rates. Nevertheless, we note that NMR measurements of metabolism in live cells or tissues are more often than not carried out without real-time monitoring of the temperature inside the NMR tube. Rather, most studies are performed using a preset determination of the temperature by correlating the temperature measured with a thermocouple inside the NMR tube (which cannot be left inside the NMR tube during the NMR measurements) and the spectrometer’s sensor for hot air that is flushed around the NMR tube. Only in recent years and with the NMR-compatible temperature probe implemented in this and other recent studies, the temperature within the NMR tube can be monitored in real-time during NMR measurements. Only in this way can the temperature variations be accurately determined and reported. We note that temperature variations cannot be fully eliminated in a system which receives hyperpolarized medium input, and that the current temperature variations are actually small and due to careful temperature regulation of the hyperpolarized medium administrated to the NMR tube containing the organoids. Nevertheless, it is warranted to further regulate the temperature and improve this aspect of the system.

Additionally, brain organoids contain large amounts of apoptotic cells. As the organoids grow larger, nutrient diffusion can become limited in the inner parts of the organoid [41]. Due to this apoptotic abundance and the small sample size in the current study, the potential negative effects of apoptosis on the metabolic analysis could not be investigated using staining for cleaved-caspase3 or other apoptotic markers. In agreement, we acknowledge that it is possible that the COs included in the current study had varying degrees of viability (and therefore NTP content per wet weight) and size of apoptotic core. For larger COs, conceivably, the diffusion of the hyperpolarized pyruvate and its ability to reach all of the organoids’ viable cells could have been compromised. Nevertheless, such are the COs, and this initial feasibility study will hopefully pave the way for further characterization of this new investigatory window into metabolism in these intact tissues.

As regards the sample size included in the current study, we believe it is sufficient for the type of feasibility study in organoids that is described herein. We note that seminal papers in the organoids literature have used small sample sizes of 2–7 organoids per age group or condition [42,43,44,45]. In the current study we have scanned altogether 15 organoids, whereas 8 organoids were scanned at the age of 10 weeks (in two batches) and 7 organoids were scanned at the age of 33 weeks (in one batch). Recapping the sample size parameters of the current experimental design: altogether, three organoids batches were investigated in 3 experimental days, each involving 2-3 hyperpolarized media injections to the same batch of organoids. An overall number of seven hyperpolarized media injections are reported with individual outcomes (Supplementary Materials, Table S1).

## Supplementary Materials

The following are available online at https://www.mdpi.com/article/10.3390/ph14090878/s1, Figure S1: Organoids in the perfusion system. Note S2. Validation of flow through the perfusion system. Figure S2. Flow of a food dye in the bypass injection system. Note S3. Signal time course and selection of time points for LDH rate determination. Figure S3. Signal time course and selec-tion of time points for LDH rate determination using the intensity of hyperpolarized [1-^13^C]pyruvate and validation with indigo food dye intensity. Table S1. Parameters and data of individual hyperpolarized medium injections included in this study. Table S2. List for the anti-bodies used in the study. Note S4. Further characterization of cerebral organoids. Figure S4.1. Validation of telencephalic identity. Figure S4.2. Ventricular zone magnification. Figure S4.3. Validation of the cellular composition of the ventricular zone.
