# Supplementary material for: Real-Time Non-Invasive and Direct Determination of Lactate Dehydrogenase Activity in Cerebral Organoids—A New Method to Characterize the Metabolism of Brain Organoids?
Source: Pharmaceuticals (Basel). 2021 Aug 30;14(9):878. doi: 10.3390/ph14090878 (PMC8465402; doi:10.3390/ph14090878)
Supplement: Supplementary file 1 [file pharmaceuticals-14-00878-s001.zip › 210902 Supplementary Materials.pdf]

Real-time non-invasive and direct determination of lactate dehydrogenase activity in cerebral organoids

Gal Sapir[1#], Daniel J. Steinberg[2#], Rami I. Aqeilan[2], and Rachel Katz-Brull[1,3*]

[1] Department of Radiology, Hadassah Medical Organization and Faculty of Medicine, Hebrew University of Jerusalem, Jerusalem Israel

[2] The Concern Foundation Laboratories, The Lautenberg Center for Immunology and Cancer Research, Department of Immunology and Cancer Research-IMRIC, Hebrew University-Hadassah Medical School, Jerusalem, Israel

[3] The Wohl Institute for Translational Medicine, Jerusalem, Israel

# Equal contribution

* Corresponding author

Supplementary Materials

S1. Organoids in the perfusion system

Photographs of organoids in the perfusion system are shown below. The filter which served for organoids positioning in the NMR tube enabled positioning of the organoids in the center of the NMR probe and the magnetic field.

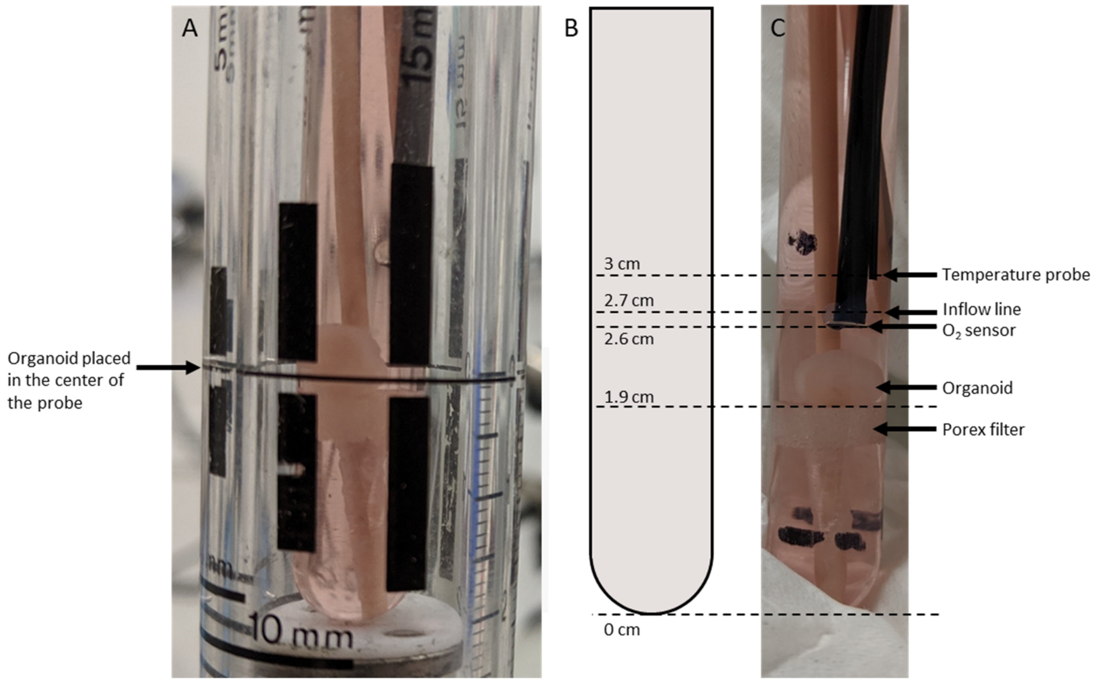

Figure S1. Organoid in the perfusion system. A) Organoid in the NMR tube, placed in the center of a 10 mm NMR probe as indicated by the NMR positioning meter. B) Schematic representation of the positioning of the lines and sensors in the NMR tube. The distances are given from the bottom of the tube. Outflow lines were placed 13 cm (main outflow line) and 15 cm (backup outflow lines) from the bottom of the tube (not shown here). C) Photograph of lines and sensors in the NMR tube. The organoid is shown on the Porex filter.

S2. Validation of flow through the perfusion system

The perfusion system used in the current experiments is shown here with injection of a dye to simulate the hyperpolarized media flow in a typical HP medium injection. It can be seen that the dye concentration (in the region seen by the NMR probe) is first increasing (wash-in, Figure S2A-B), then plateauing (constant concentration, Figure S2C-F), then decreasing (wash-out, Figure S2G-J). The dye was completely washed out from the probe region 4 min after the start of the injection (Figure S2J). The same timings could be observed when investigating the signal of hyperpolarized [1-^{13}C]pyruvate corrected for an effective decay due to RF pulsation and T_1 relaxation (Figure S3). Only timepoints for which the concentration of hyperpolarized [1-^{13}C]pyruvate was constant (14 mM) were analyzed for rate-determination.

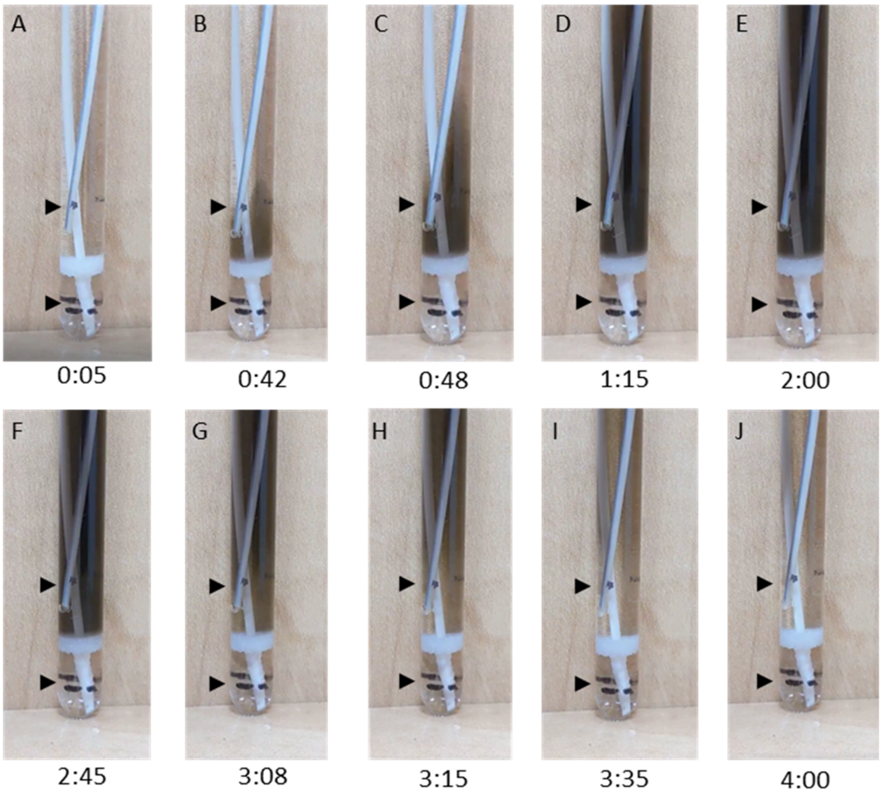

Figure S2. Flow of a food dye in the bypass injection system. Temporal dynamics of the bypass injection system, with dye representing hyperpolarized media. Times are represented as min:s. 0:00 is the time when the 3-way valves were adjusted to allow flow through the bypass. In this experiment, the peristaltic pump was calibrated to operate at a rate of 4.2 mL/min. A) Before arrival of the dye to the probe region, B) wash-in of the dye into the probe region started, C) dye reached a steady level in the probe area, D-F) dye remains in the probe area, G) washout of dye started, H) the probe region is cleared of dye, I-J) washout of dye from the probe region is completed. The total time in which the dye is in the plateau phase (constant concentration) is approximately 2.5 min. Black arrowheads mark the sensitive region of the NMR probe.

S3. Signal time course and selection of time points for LDH rate determination

Figure S3 shows the time course of hyperpolarized [1-^{13}C]pyruvate signal following injection to cerebral organoids, with an overlapping time course of the intensity of water-soluble indigo food dye (shown in Figure S2). The of hyperpolarized [1-^{13}C]pyruvate signal was corrected with a T_{1_eff} calculation as described equation (1)

$$S_{Pyr_{corr}}(t) = S_{Pyr}(t) * e^{(\frac{t}{T1\_eff})} \quad (1)$$

The value of T_{1_eff} was chosen experimentally to be 57 s as previously described [1]. Indeed, this factor was able to correct the hyperpolarized [1-^{13}C]pyruvate decay curve to display the flow characteristics of the current perfusion system (shown in Figure S3) for all the injections included in the current study. The duration of constant hyperpolarized [1-^{13}C]pyruvate concentration in the NMR tube was determined as the time points which showed a corrected signal of hyperpolarized [1-^{13}C]pyruvate that was no less than 80 % of the maximal point (of the T_{1_eff} corrected signal). This time duration was about 120 s per injection. The signals of [1-^{13}C]lactate obtained during this time window were used for the calculation of LDH rate. The intensity of food dye was determined in 1 s intervals from a video of the working perfusion system (still images from this video are shown is Figure S2).

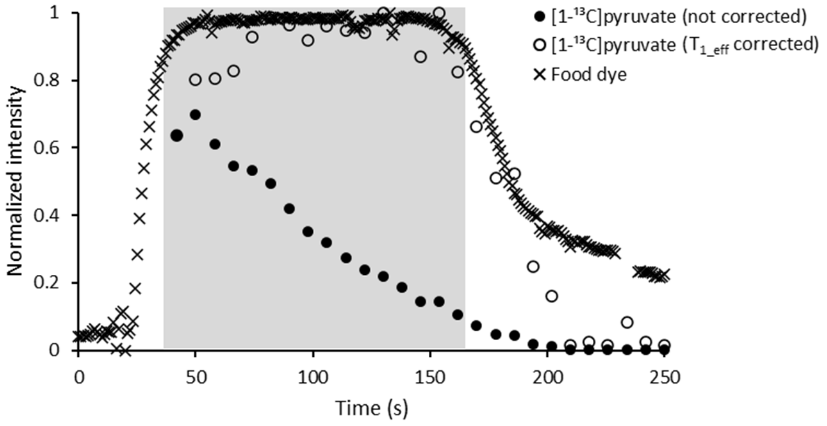

Figure S3. Signal time course and selection of time points for LDH rate determination using the intensity of hyperpolarized [1-^{13}C]pyruvate and validation with indigo food dye intensity. A typical time course of the hyperpolarized signal intensity of [1-^{13}C]pyruvate following an injection to an NMR tube containing COs is shown with full circles (measured signal, mostly decays), and open circles (T_{1_eff} corrected signal). The corrected signal is indicative of the concentration of [1-^{13}C]pyruvate in the sensitive region of the NMR probe. The shaded area marks the duration at which the concentration of both hyperpolarized [1-^{13}C]pyruvate and the food dye were constant and maximal. Only measurements within this time frame were used for the enzymatic rate determination (Methods). At t=0, the hyperpolarized substrate or the food dye started flowing from the bypass into the NMR tube. Timepoints 238-240 s of the food dye data were omitted due to technical problems with the movie. Color intensity analysis was performed as follows: individual frames at 1 s resolution were extracted using the FFmpeg

software (version 4.3.1, FFmpeg team, https://ffmpeg.org/). Color analysis in the region visible to the NMR probe was performed using the OpenCV software library (version 4.5.1, Intel, Santa Clara, CA, USA, https://opencv.org/).

*Table S1. Parameters and data of individual hyperpolarized medium injections included in this study**

Organoid age (weeks)	Number of organoids	Total wet weight (mg)	Hyperpolarized medium injection number**	NTP content before the injection (µmol)	Average LDH activity (nmol/s)
10	3	20.3	1	0.21	0.46
			2	0.16	0.68
10	5	76.6	1	0.10	3.09
			2	0.10	2.21
			3	0.10	1.73
33	7	58.8	1	0.09	1.04
			2	0.10	1.06

*The experiments are listed in the order in which they were performed. ** For each experimental day (organoids batch).

Table S2. List for the antibodies used in the study

Antibody	Host	Supplier	Catalog number	Dilution
TUBB3 (TUJ1)	Mouse	BioLegend[1]	801202	1:1000
SOX2	Rat	Invitrogen[2]	14-9811-80	1:1000
S100β	Mouse	Abcam[3]	ab11178	1:400
Ki67	Rabbit	Invitrogen[2]	MA5-14520	1:200
Anti-mouse Alexa Fluor 488	Goat	Invitrogen[2]	A11029	1:1000
Anti-rabbit Alexa Fluor 647	Goat	Invitrogen[2]	A21244	1:1000
AffiniPure Anti-Rat Cy5	Donkey	Jackson ImmunoResearch[3]	712-175-150	1:1000
NeuN	Mouse	Sigma-Aldrich[4]	MAB377	1:400
CRYAB	Mouse	Abcam[3]	ab13496	1:250
PAX6	Rabbit	Covance[5]	PRB-278P	1:350
TBR2	Rabbit	Abcam[3]	ab2283	1:300

[1] San Diego, CA, USA
[2] Carlsbad, CA, USA
[3] Cambridge, UK
[4] Rehovot, Israel
[5] Princeton, NJ, USA

S4. Further characterization of cerebral organoids.

Figures S4.1 to S4.3 demonstrate additional immunohistochemical staining of COs that confirmed their telencephalic identity and the composition of the ventricular zone. COs were fixated and cryosectioned in weeks 5 (Figure S4.1) and 10 (Figures S4.2, S4.3). Week 5 COs were stained for PAX6, confirming telencephalic identity. PAX6 positive cells are surrounded by TUJ1 positive cells (class III β-tubulin, a marker of early-born neurons). CRYAB, a marker for ventricular radial glia (vRGs) was used to validate the identity of SOX2 positive cells in the ventricular zone. Week 10 COs were stained for TBR2, a marker for intermediate progenitors and NeuN, a marker for mature neurons.

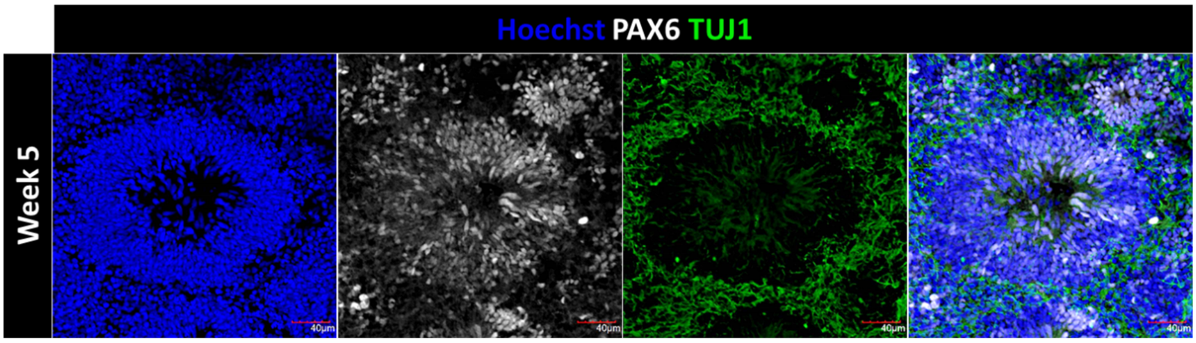

Figure S4.1. Validation of telencephalic identity. Week 5 COs stained for the dorsal progenitor marker PAX6 and for neuron-specific class III β-tubulin (TUJ1), demonstrating the acquisition of telencephalic identity (n=2 organoids).

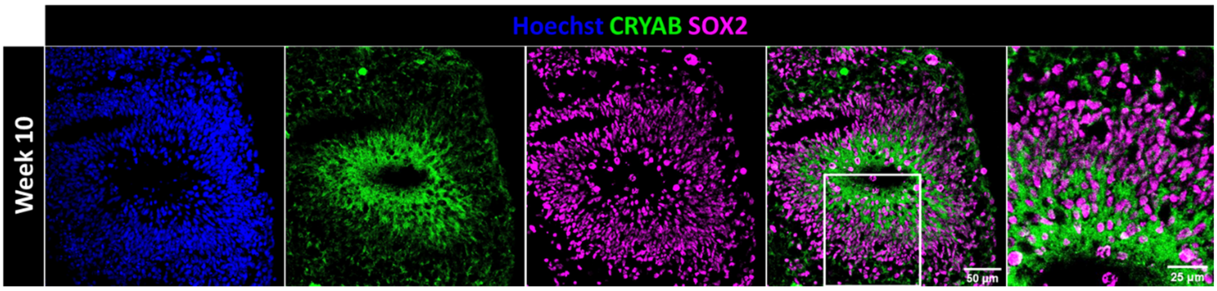

Figure S4.2. Ventricular zone magnification. Week 10 COs stained for the ventricular radial glia (vRGs) marker CRYAB. The image on the right is an enlargement of the boxed area (upside down). Scale = 25 µm (n=4 organoids).

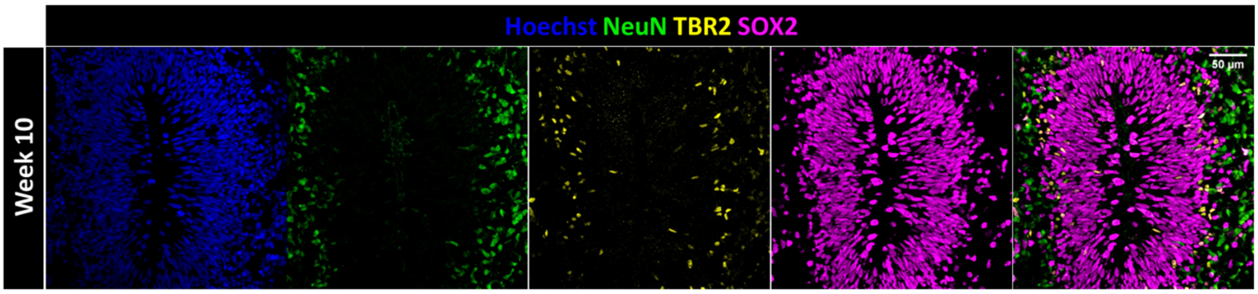

Figure S4.3. Validation of the cellular composition of the ventricular zone. Week 10 COs stained for the mature neural marker NeuN, intermediate neurons marker TBR2 (Eomesodermin), and the progenitor marker SOX2 (n=4 organoids).

Reference

1. Sapir G, Shaul D, Lev-Cohain N, Sosna J, Gomori MJ, Katz-Brull R. LDH and PDH activities in the ischemic brain and the effect of reperfusion—An ex vivo mr study in rat brain slices using hyperpolarized [1-13C]pyruvate. *Metabolites.* 2021; 11(4): 210.
